# Complete genome assembly and methylome dissection of *Methanococcus aeolicus* PL15/H*^p^*

**DOI:** 10.3389/fmicb.2023.1112734

**Published:** 2023-04-05

**Authors:** Alexey Fomenkov, Peter Weigele, Colleen McClung, Casey Madinger, Richard J. Roberts

**Affiliations:** New England Biolabs Inc., Ipswich, MA, United States

**Keywords:** complete genome, methylome, *Methanococcus aeolicus* PL15/H*^p^*, restriction, modification, systems

## Abstract

Although restriction-modification systems are found in both Eubacterial and Archaeal kingdoms, comparatively less is known about patterns of DNA methylation and genome defense systems in archaea. Here we report the complete closed genome sequence and methylome analysis of *Methanococcus aeolicus* PL15/H*^p^*, a strain of the CO_2_-reducing methanogenic archaeon and a commercial source for *Mae*I, *Mae*II, and *Mae*III restriction endonucleases. The *M. aeolicus* PL15/H*^p^* genome consists of a 1.68 megabase circular chromosome predicted to contain 1,615 protein coding genes and 38 tRNAs. A combination of methylome sequencing, homology-based genome annotation, and recombinant gene expression identified five restriction-modification systems encoded by this organism, including the methyltransferase and site-specific endonuclease of *Mae*III. The *Mae*III restriction endonuclease was recombinantly expressed, purified and shown to have site-specific DNA cleavage activity *in vitro*.

## Introduction

The role of DNA methylation in the restriction-modification (RM) mechanisms of bacteria and archaea was discovered more than 50 years ago (Arber, 1974; Loenen et al., 2014). Bacterial and archaeal DNA methyltransferases (MTases) create an epigenetic landscape in DNA by modifying specific motifs and, in concert with their cognate restriction endonucleases, determine the boundaries between bacterial species and protect against the entry of bacteriophages, conjugative plasmids and other mobile DNA elements. In addition to their roles in specifying self/non-self during genome defense, DNA methyltransferases appear as standalone genes; i.e., not associated with restriction endonucleases. Such “orphan” methyltransferases take on epigenetic functions such as regulation of genes for pathogenesis (Hernday et al., 2003) and ensuring fidelity of DNA repair (Schlagman et al., 1986).

Compared to their bacterial counterparts, the RM systems of archaea are relatively understudied. For example, of 54,450 genomes currently known to harbor RM systems, only 613 are from archaea (Roberts et al., 2022). Three restriction endonucleases, *Mae*I (C↓TAG), *Mae*II (A↓CGT), and *Mae*III (↓GTNAC) have previously been characterized from *Methanococcus aeolicus* PL15/H*^p^* (Schmid et al., 1984). *M. aeolicus* PL15/H*^p^*, originally isolated from marine sediments of Lipari Island near Sicily (Kendall et al., 2006), is a strain of CO_2_-reducing methanogenic Euryarchaeota. Because the genome sequence of this strain has not been reported, we decided to perform SMRT sequencing of this archaeon to identify the Restriction-Modification (RM) system genes. Next generation sequencing platforms such as Single Molecule Real Time (SMRT) (Clark et al., 2012), Rapid Identification Methylase Specificity (RIMS) (Baum et al., 2021), and Oxford Nanopore Technology (ONT) (Tourancheau et al., 2021) allow not only the sequence and complete assembly of bacterial and archaeal genomes, but also enable the determination of methylation patterns and the confirmation of the MTase genes responsible for the modified motifs.

Herein, we report the genome sequence and methylome of *Methanococcus aeolicus* PL15/H*^p^*, results which enabled the identification of genes encoding five RM-systems. Of these, methyltransferase activities of M.MaeIV and M.MaeV, as well as the restriction endonuclease activity of *Mae*III, were further verified and characterized experimentally. These findings reveal the genetic basis of commercially relevant restriction endonucleases, connect methyltransferase genes to the epigenetic state of *M. aeolicus*, and extend what is known about RM systems in the Methanococcales branch of the Euryarchaeal lineage.

## Materials and methods

### Strains, plasmids, and reagents

Cells of *Methanococcus aeolicus* PL15/H*^p^* were a gift from Prof. Dina Grohmann (Universität Regensburg), grown as previously described (Kendall et al., 2006). All laboratory *Escherichia coli* strains used in this study are listed in Supplementary Table 1. Cultures were grown in Luria-Bertani (LB) broth or agar on appropriate antibiotics. All plasmids used in this study are listed in Supplementary Table 2. Restriction enzymes, except *Mae*III (Roche Diagnostic, GmbH, Mannheim, Germany), DNA substrates, DNA and protein markers were from New England Biolabs (NEB, MA). Q5 “Hot Start” DNA polymerase (M0543, NEB, MA) was used for PCR amplification of Methyltransferase and Restriction endonuclease genes for cloning. All PCR primers were synthesized by IDT, IA and are listed in Supplementary Table 3. The cloning of PCR amplified genes in appropriate vectors were performed using NEBuilder HiFi DNA assembly Master mix (E2621, NEB, MA). The plasmid DNA was purified using a Monarch Plasmid Miniprep kit (T1010, NEB, MA).

### SMRT sequencing and genome assembly

Genomic DNA from *Methanococcus aeolicus* PL15/H*^p^* was purified using a Monarch Genome Purification kit (T3010, NEB, Ipswich, MA, USA) and the DNA sample was sheared to an average size of ∼ 10 kb using the G-tube protocol (Covaris, Woburn, MA, USA). DNA libraries were prepared using a SMRTbell express template prep kit 2.0 (100–938–900, Pacific Biosciences, Menlo Park, CA, USA) and ligated with hairpin barcoded adapter lbc1-lbc1. Incompletely formed SMRTbell templates were removed by digestion with a combination of exonuclease III and exonuclease VII (NEB, Ipswich, MA, USA). The qualification and quantification of the SMRTbell libraries were made on a Qubit fluorimeter (Invitrogen, OR) and a 2,100 Bioanalyzer (Agilent Technologies, Santa Clara, CA, USA). SMRT sequencing was performed using an SQ1 (Pacific Biosciences, Menlo Park, CA, USA) based on the multiplex protocol for 10 kb SMRTbell library inserts. Sequencing reads were collected and *de novo* assembled using the Microbial Assembly version 10.1.0.1119588 program with default quality and read length parameters. In addition to genome assembly (Chin et al., 2013), the SMRT Analysis pipeline from Pacific Biosciences^1^ enables the determination of the epigenetic status of sequenced DNA by identifying the m6A and m4C modified motifs (Flusberg et al., 2010; Clark et al., 2012; Korlach and Turner, 2012).

### Computational and experimental identification of *M. aeolicus* PL15/H*^p^* methyltransferases

Following an approach nicknamed the “Hungarian trick” (Szomolányi et al., 1980), 5 μg of *M. aeolicus* PL15/H*^p^* gDNA was digested with *Bam*HI, *Bgl*II and partially digested with *Sau*3AI restriction enzymes. DNA fragments were cloned into the compatible dephosphorylated *Bam*HI sites of pUC19, pBR322 and pACYC184 vectors yielding 9 shot gun libraries. The foreign restriction and modification genes are usually very toxic for *E. coli*, therefore in order to clone both we need high level of methylation. These three vectors have different copy numbers pUC19 (high), pBR322 (medium) and pACYC184 (low). Moreover, they utilized different promoters P_*lac*_ and P_*tet*_, since native *Methanococcus* promoters may or may not work in *E. coli*. Each purified plasmid library (1 μg) was challenged with 10 units of *Mae*III restriction endonuclease and the digestion mixtures were transformed into *E. coli* strain ER2683. Plasmids were recovered from surviving transformants and analyzed by Sanger sequencing and restriction digest.

Homology-based searches of the *M. aeolicus* genome for putative methyltransferase genes were performed using the Seqware program (Roberts et al., 2022). Additional searches for MTase genes were performed using HMMer (HMMER 3.3.2)^2^ to annotate each predicted protein coding sequence feature (CDS) of the *Methanococcus aeolicus* PL15/H*^p^* genome. Genes matching MTase sequence profiles were further examined by structure prediction using the ColabFold implementation of AlphaFold2 (Jumper et al., 2021; Mirdita et al., 2022), followed by structure similarity search using predicted MTase models as query inputs to DALI (Holm, 2022).

Gene loci for M.MaeV and M1-M2.MaeIV were PCR amplified with P1-P2 primers and P3-P4 primers, respectively, and assembled into low copy pACYCΔtet vectors under control of a constitutive P_*tet*_ promoter and expressed in *E. coli* strain ER2683 (Supplementary Table 1). The correct clones were verified by Sanger sequencing of the inserts using an ABI DNA sequencer with pTetF and pTetR sequencing primers and transformed into the methylation deficient *E. coli* strain ER2796 (Supplementary Table 1).

### Liquid chromatography-tandem mass spectrometry analysis of functionally purified *Mae*III endonuclease

Purified *Mae*III endonuclease was subjected to 10–20% Tris-Glycine SDS-PAGE and stained with SimplyBlue SafeStain (Invitrogen, OR). The major band at approximately 35 kDa was excised for in-gel digestion (Figure 4A). The gel band was sliced into 1 mm^3^ pieces and the pieces were divided into two aliquots for multiple enzymatic digestions (Wiśniewski et al., 2019). The gel pieces were destained, and the protein was reduced with DTT, alkylated with iodoacetamide, and then digested with either Trypsin-ultra, Mass Spectrometry Grade (P8101; NEB, Ipswich, MA, USA) or subtilisin (Sigma-Aldrich, St. Louis, MO, USA) at a protease concentration of 2 ng/μL. Digestions were conducted for 1 h (50°C for trypsin, 37°C for subtilisin) and were quenched with trifluoroacetic acid at a final concentration of 0.5% for downstream analysis.

The resulting peptides were loaded *via* Proxeon Easy-nLC 1,200 (ThermoFisher, Waltham, MA, USA) onto a reversed phase analytical column 25 cm × 100 μm ID, Reprosil 3 μm C18 (New Objective, Littleton, MA, USA) and eluted at a flow rate of 0.3 μL/min over a 35 min gradient of organic solvent (mobile phase A: 0.1% formic acid in water; mobile phase B: 90% acetonitrile/0.1% formic acid) as follows: 1-min equilibration at 10% B, 10–40% B in 25 min, 40–50% B in 4 min, and 50–78% B in 5 min. Peptides were electro-sprayed into a LTQ Orbitrap XL with a Nanospray Flex Ion Source (Thermo Fisher, Waltham, MA, USA) and fragmentation spectra were generated by collision-induced dissociation (CID). Full scans between 400–1,600 m/z were acquired with 30 k resolution. MS/MS spectra were generated in a data-dependent manner, with a normalized collision energy of 35.0 and dynamic exclusion of 30 s. The five most intense ions (including + 1 charge states for subtilisin-generated peptides) were selected for fragmentation.

Acquired MS/MS spectra were analyzed using PEAKS Studio Xpro (Bioinformatics Solutions, Inc., Waterloo, ON, USA). The data were searched against *Methanococcus aeolicus* PL15/H*^p^* translated ORFs from the genome sequence, allowing for a fixed carbamidomethyl modification on cysteines, variable oxidation of methionine, and two missed cleavages (tryptic peptides). The precursor and fragment mass tolerances were set to 20 ppm and 0.6 Da, respectively. Identified proteins were filtered by a 1% false discovery rate and required at least two unique peptides per protein.

### Protein purification and endonuclease assay

The recombinant *Mae*III restriction enzyme was partially purified on Ni-NTA magnetic beads (S1423L, NEB, Ipswich, MA, USA) according to the manufacturer’s protocol. DNA digestion patterns obtained by titration of restriction endonuclease activity from 250 mM imidazole Ni-NTA column elution fraction from *E. coli* clone 3081[pM.*Mae*III + pET21bMaeIII] expressing *Mae*III enzyme compared to *Mae*III restriction enzyme from Roche on the same DNA substrate on λ DNA substrate. 1 μg of λ DNA incubated with Ni-NTA imidazole elution fraction of 6×His:*Mae*III serial 3× dilution (from 9 to 0.1 μl) and serial 3× dilution of commercial *Mae*III enzyme (from 9 to 0.1 units) for 1 h at 55°C in 1× *Mae*III commercial buffer (Roche Diagnostic, GmbH, Mannheim, Germany). 2× Buffer contains 40 mM Tris–HCl, 550 mM NaCl, 12 mM MgCl_2_, 14 mM 2-mercaptoethanol, pH 8.2 (+55°C). The reaction mixtures were separated on 0.8% agarose gel in TBE buffer and visualized by staining with ethidium bromide.

## Results

### DNA-sequencing and genome assembly

**FIGURE 1:**
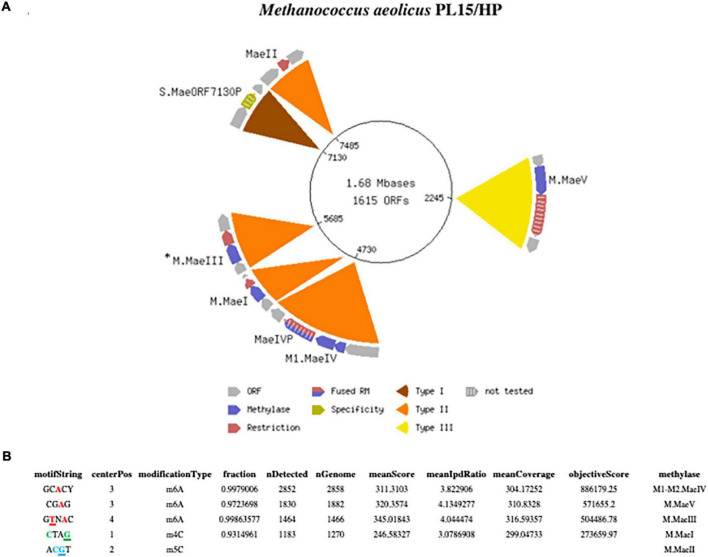
**(A)** Schematic presentation of the complete closed circular genome of *Methanococcus aeolicus* PL15/H*^p^* showing the location of the RM systems. The M.*Mae*III gene was not directly identified by the Seqware program (*). **(B)** Detection of modified DNA motifs identified by SMRT sequencing in *M. aeolicus* PL15/H*^p^* genome. Note that 5mC methylation by the M.*Mae*II gene was not directly identified by SMRT sequencing.

Monarch purified gDNA (2 μg) from a *Methanococcus aeolicus* PL15/H*^p^* culture was used to make one barcoded (lbc1-lbc1) 10 kb SMRT bell library sequenced on an SQ1 instrument for 20 h. 418,400 long continuous sequencing subreads with 2,583 bp mean subread lengths were converted to 12,577 HiFi subreads with 5,328 bp mean subread length, yielding 43.7 Mb of high-quality sequencing data. The polished assembly generated one closed circular genomic element of 1,677,738 bp with 26-fold genome coverage and 29.93% G + C content for the chromosome (Figure 1A). The assembled sequence was annotated using the National Center for Biotechnology Information (NCBI) Prokaryotic Genome Annotation Pipeline (PGAP) (Tatusova et al., 2016; Haft et al., 2018) which identified 1,615 protein coding features and 38 tRNA genes. The annotated genome sequence of *M. aeolicus* PL15/H*^p^* is available from NCBI *via* the Bioproject PRJNA622823; BioSample: SAMN30825808, SRA: SRS15238210, and genome sequence: NZ_CP104873.

### SMRT motif and modification analysis of *Methanococcus aeolicus* PL15/H*^p^* genome

One advantage of the SMRT sequencing platform is its ability to directly detect the epigenetic state of sequenced DNA. As the DNA sequencing polymerase traverses methylated nucleobases in the sequencing template, changes in the amount of time between the nucleotide incorporation events (also known as the Interpulse Duration, or IPD) are recorded and analyzed by the SMRT-Link software yielding m6A and m4C modification motifs (Flusberg et al., 2010; Clark et al., 2012). The complete motif and modification analysis of assembled *Methanococcus aeolicus* PL15/H*^p^* using these kinetic signatures detected three m6A and one m4C modified motifs (Figure 1B). Two of the motifs with m4C **C**TAG and m6A CTN**A**G were recognized as signatures of the previously discovered *Mae*I and *Mae*III RM systems (Schmid et al., 1984). The m5C motif of *Mae*II system A**C**GT cannot be directly detected by SMRT sequencing due to the weak IPD ratio signal generated by DNA polymerase on m5C modified base. Two additional m6A motifs with single DNA strand modification GC**A**CY and CG**A**G were also detected by SMRT motif and modification analysis.

### Matching putative DNA MTase genes to appropriate motifs

**FIGURE 2:**
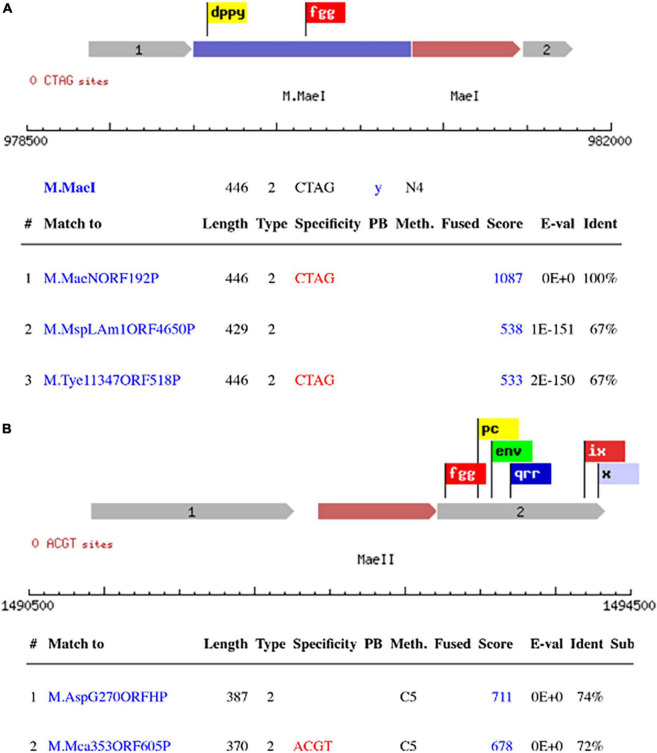
Schematic presentation of RM systems assigned bioinformatically based on homology search against DNA methylase genes with known specificity in REBASE. M.*Mae*I with the top 3 blast hits **(A)**, M.*Mae*II with the 2 top blast hits **(B)**. The flags represent the conserved motifs that are used to identify the methyltransferase genes.

**FIGURE 3:**
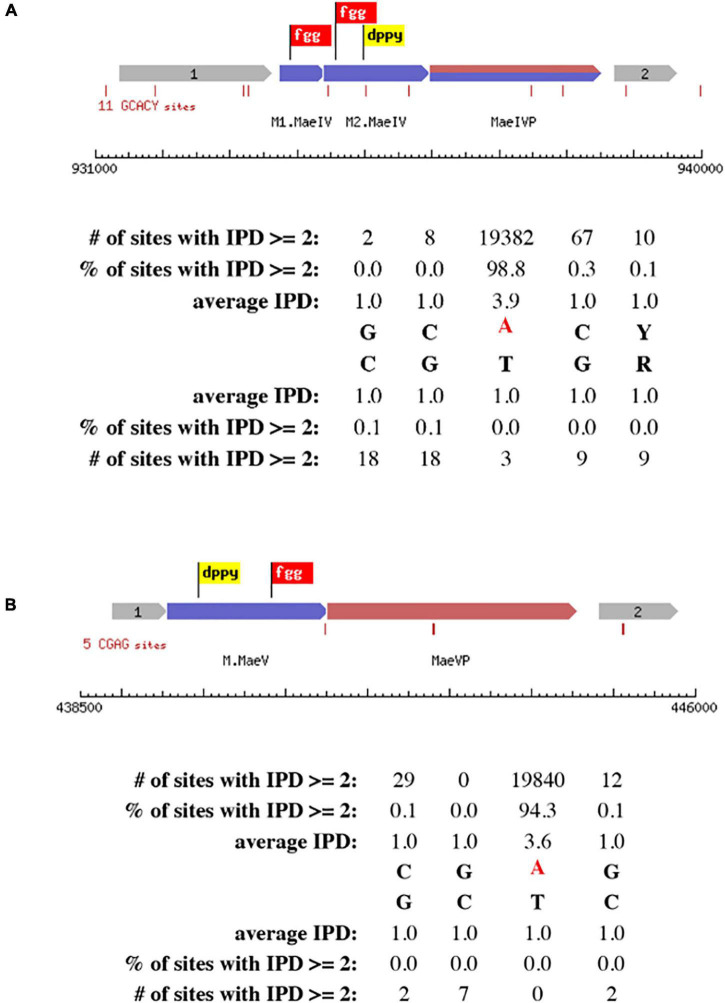
Schematic presentation of RM systems assigned experimentally by cloning and SMRT motif and modification analysis. The correct motifs were verified by analyzing SMRT kinetic data using MotStat software (Vincze, unpublished) for M.MaeIV **(A)** and M.MaeV **(B)**. The flags represent the conserved motifs that are used to identify the methyltransferase genes.

The primary scanning of the *M. aeolicus* PL15/H*^p^* assembled genome using Seqware identified 4 putative methyltransferase genes. Two of them for M.*Mae*I and M.*Mae*II where assigned bioinformatically based on homology search against methyltransferase genes in REBASE with known specificities (Figures 2A, B). The two other putative methyltransferase genes were assigned experimentally. Gene loci for M.MaeV and M1-M2.MaeIV were cloned into low copy pACYCΔtet vectors under the control of a constitutive P_*tet*_ promoter and their expression induced in *E. coli* strain ER2683 (Supplementary Table 1). SMRT sequencing and methylation analysis of *E. coli* gDNA recovered from pM1-2.MaeIV and pM.MaeV expressing clones revealed that M.MaeV recognized the m6A modified motif CG**A**G. M1.MaeIV and M2.MaeIV belong to a family of so called split methylase/RM genes similar to DrdVI (Fomenkov et al., 2019) and recognize the m6A GC**A**CY modified motif (Figures 3A, B). However, at that point, we were not able to assign the m6A GTN**A**C motif, corresponding to M.*Mae*III, as there was no candidate gene identified by the PGAP annotation.

### Searching for the *Mae*III restriction-modification system

To find the missing gene for M.*Mae*III we decided to apply an old methylation selection method known as the “Hungarian trick” (Szomolányi et al., 1980). Basically, this method is based on selection of clone(s) expressing the methyltransferase by treatment of the library with the cognate restriction endonuclease to destroy unmodified plasmid DNAs, leaving only plasmids that contain an active methyltransferase gene. In addition, we made a fosmid library in the pCC1Fos vector (Epicenter, WI). The plasmid and fosmid DNAs from survival clones were tested using a methylation protection assay for *Mae*III resistance to digestion. Unfortunately, after selection and screening we were not able to find any *Mae*III resistant clones (data not shown). Therefore, we decided to use a different approach to find the missing genes.

**FIGURE 4:**
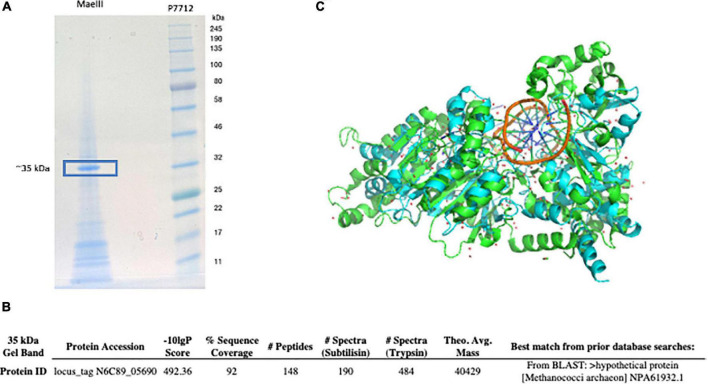
*Mae*III restriction endonuclease separated on 10–20% Tris-Glycine SDS-PAGE and stained with SimplyBlue SafeStain. The protein band at ∼ 35 kD was excised and used for MS/MS analysis **(A)**. The sequence matched locus_tag N6C89_05690 from the *M. aeolicus* PL15/H*^p^* genome was detected by MS/MS analysis as putative *Mae*III restriction enzyme **(B)**. *M. aeolicus* PL15/H*^p^* assembled genome was scanned for additional DNA methyltransferase genes using an automated implementation of HMMer, which produced another potential hit corresponding to locus_tag N6C89_5685. The predicted structure of the M.*Mae*III protein through AlphaFold2 monomeric modeling **(C)**. The best scoring match to an AlphaFold2 predicted model of N6C89_5685 locus was 7lt5, the PDB structure of CamA, an adenine methyltransferase of *Clostridioides difficile* (Zhou et al., 2021).

We used an MS/MS method to identify the protein sequence in functionally purified *Mae*III from Roche (Figure 4A). This yielded a top target hit corresponding to locus_tag N6C89_05690 from *M. aeolicus* PL15/H*^p^* (Figure 4B). In addition, the *M. aeolicus* PL15/H*^p^* assembled genome was scanned for additional DNA methyltransferase genes using an automated implementation of HMMer, which produced another potential hit corresponding to locus_tag N6C89_5685. The best scoring match to an AlphaFold2 predicted model of N6C89_5685 was 7lt5, the PDB structure of CamA, an adenine methyltransferase of *Clostridioides difficile* (Figure 4C; Zhou et al., 2021). Thus, the close proximity of the two identified target genes strongly suggested that they corresponded to the *Mae*III RM system.

### Cloning and expression of the *Mae*III restriction-modification system

**FIGURE 5:**
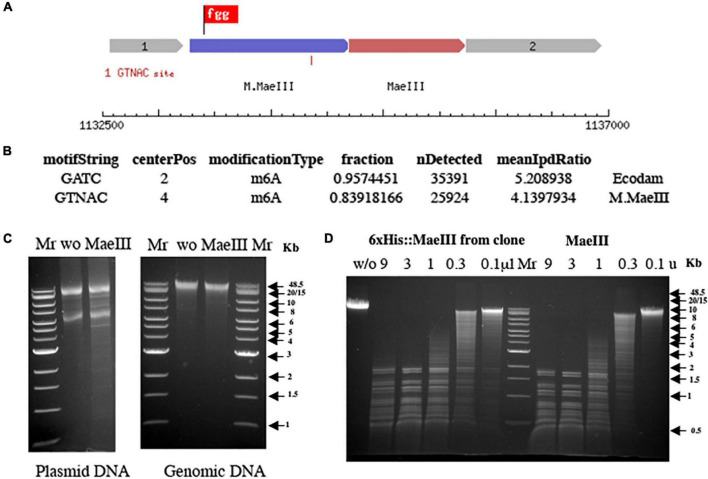
Schematic presentation of the *Mae*III restriction-modification system **(A)**. Motif and modification analysis by SMRT sequencing of gDNA from *E. coli* strain ER3081 [pM.*Mae*III] **(B)**. Note that this strain contains a Dam MTase in addition to M.*Mae*III. *Mae*III methylation protection assay on both plasmid and gDNA from *E. coli* 3081[pM.*Mae*III] strain **(C)**. Plasmid and gDNA from *E. coli* 3081[pM.*Mae*III] were purified and 1 μg of each DNA was incubated without (w/o) as a negative control or with 1 unit of *Mae*III restriction enzyme at 55°C for 1 h in 1× *Mae*III commercial buffer (Roche Diagnostic, GmbH, Mannheim, Germany). The results demonstrated that both plasmid, and gDNA are resistant to *Mae*III restriction endonuclease digestion due to modification of the DNA *in vivo* by M.*Mae*III methyltransferase at GTNAC sites. λ DNA digestion patterns obtained by titration of restriction endonuclease activity from 250 mM imidazole Ni-NTA column elution fraction from *E. coli* clone 3081[pM.*Mae*III + pET21b 6×His:*Mae*III] expressing *Mae*III enzyme compared to *Mae*III restriction enzyme from Roche on the same DNA substrate **(D)**. 1 μg of λ DNA incubated with Ni-NTA imidazole elution fraction of 6×His:*Mae*III serial 3× dilution (from 9 to 0.1 μl) and serial 3× dilution of commercial *Mae*III enzyme (from 9 to 0.1 units) for 1 h at 55°C in 1× *Mae*III commercial buffer (Roche Diagnostic, GmbH, Mannheim, Germany). 2× Buffer contains 40 mM Tris–HCl, 550 mM NaCl, 12 mM MgCl_2_, 14 mM 2-mercaptoethanol, pH 8.2 (+55°C). The reaction mixtures were separated on 0.8% agarose gel in TBE buffer and visualized by staining with ethidium bromide.

The genes corresponding to locus_tags 5,685 and 5,690 (Figure 5A) were cloned into the low copy pACYCΔtet vector under control of a constitutive P_*tet*_ promoter for expression in *E. coli* T7 expression strain, ER3081. The plasmid and gDNA from this strain were purified and challenged with 10 units of the *Mae*III restriction enzyme to test for methylation protection (Figure 5C). In addition, the DNA obtained from *E. coli* expressing this putative M.*Mae*III methyltransferase was sequenced using the SMRT analysis platform, identifying the sequence GTNAC as modified (Figure 5B), consistent with the M.*Mae*III modification motif previously reported (Schmid et al., 1984). These results clearly indicated that locus_tag N6C89_5685 did indeed code for M.*Mae*III. Therefore the strain was designated as ER3081[pM.*Mae*III].

A PCR amplicon spanning the presumptive endonuclease gene, locus_tag 5690, was cloned in-frame with the N-terminal 6× His tag and linker sequence of the pET21b expression vector under the control of the pT7 promoter and transformed into an *E. coli* strain previously transformed with a compatible M.*Mae*III expressing plasmid. The resulting two-plasmid strain encoding M.*Mae*III and the putative *Mae*III endonuclease gene was induced and the his-tagged putative endonuclease purified using Ni-NTA beads as described. *Mae*III restriction endonuclease activity was subsequently detected in a 250 mM imidazole elution fraction (Figure 5D).

## Discussion

In this paper we report the complete closed genome of *Methanococcus aeolicus* PL15/H*^p^*. The sequencing data are publicly available from NCBI. In addition, the complete methylome of this strain and the identified RM systems may help other investigators to develop novel genetic systems for *M. aeolicus* and create efficient transformation protocols for this archaeal species.

**FIGURE 6:**
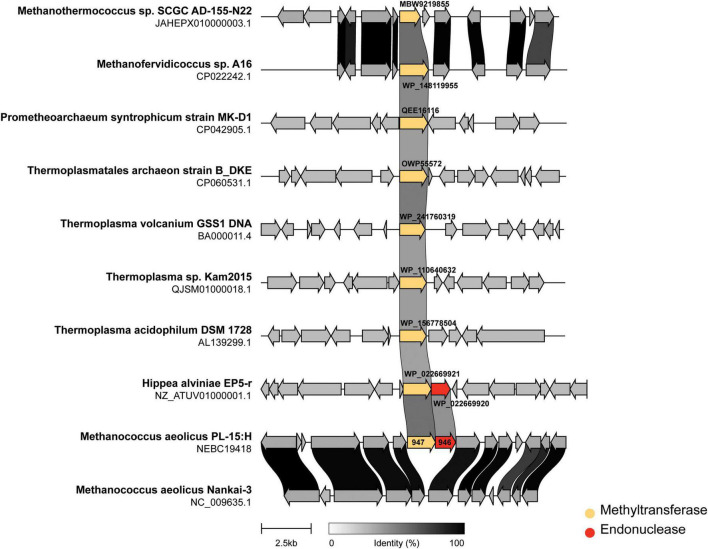
Genomic neighborhoods of M.*Mae*III homologs across representative archaea. The m6A DNA methylase (M.*Mae*III) of *M. aeolicus* PL-15:H was used as a query sequence for a BLAST search taxonomically limited to archaea (taxid:2,157) at NCBI. Eight genomes from cultured archaea and one bacterium containing matching sequences to M.*Mae*III with an *e*-value of less than 10^–30^ were selected for genome neighborhood analysis. Sub genomic sequences surrounding the M.*Mae*III homologs were manually obtained by computationally extracting 7.5 kb of sequence, plus annotations, upstream and downstream of the methyltransferase encoding sequence. The resulting gene clusters were aligned and rendered using “clinker” gene diagramming software (Gilchrist and Chooi, 2020) with the methyltransferase colored yellow and endonuclease colored in red. The degree of amino acid identity among coding sequences are indicated by grayscale links connecting homologous genes.

In addition we performed genomic neighborhood analysis of M.*Mae*III homologs across representative archaea. Figure 6 shows the methyltransferases (indicated in yellow) either in an apparent “orphan” configuration (i.e., no neighboring restriction endonuclease) or associated with an endonuclease sequence (indicated in red). *M. aeolicus* PL-15:H and *M. aeolicus* Nankai-3 are highly syntenic at this locus, however, *M. aeolicus* Nankai-3 completely lacks the *Mae*III RM system. Like the Nankai-3 strain, *M. jannaschii* lacks a M.*Mae*III homolog. An *Mae*III-like RM system is found in the thermophilic bacterium *Hippea alviniae* EP5-r. M.*Mae*III homologs are found in a variety of unrelated contexts across representatives from Euryarchaeota and Lokiarchaeota. One conserved configuration of an M.*Mae*III-like gene occurs between *Methanothermococcus* sp., SCGC AD-144-N22 and *Methanofervidicoccus* sp., A16 where apparent orphan methylases are found in syntenic loci sharing a high degree of amino-acid identity. The function of these orphan methyltransferases is currently unknown.

Taken together, the data presented herein extend what is known about the occurrence and function of RM systems in the Methanococcales branch of the Euryarchaeota lineage. The synteny/neighborhood analysis presented in Figure 5 are consistent with previously reported findings in the Haloarchaea (Fullmer et al., 2019); i.e., the *Mae*III RM system does not seem to be conserved between closely related *Methanococcus* species and yet is found intact in a eubacterial extremophile. These observations are suggestive of a mobile and dynamic distribution not only of the intact RM system, but also homologous methyltransferases found in “orphan” configuration. It remains to be seen what, if any, would be the impacts of the RM systems and orphan methyltransferases of *Methanococcus* on mating (Makkay et al., 2020), transformability (Zatopek et al., 2021), and DNA repair.

## Data availability statement

The datasets presented in this study can be found in online repositories. The names of the repository/repositories and accession number(s) can be found below: https://www.ncbi.nlm.nih.gov/genbank/, CP104873.

## Author contributions

AF performed SMRT sequencing, genome assembly, and writing—original manuscript. PW performed bioinformatic analysis. CMc and CMa performed MS/MS analysis. RR performed methylome analysis and editing final manuscript. All authors contributed to the article and approved the submitted version.

## Footnotes

1
http://www.pacbiodevnet.com/SMRT-Analysis/Software/SMRT-Pipe

2
http://hmmer.org/
